# Development and Evaluation of a Patient–Family Caregiver Dyad mHealth Intervention for Heart Failure Self-Care: Quasi-Experimental Study

**DOI:** 10.2196/74922

**Published:** 2025-06-16

**Authors:** Youn-Jung Son, JiYeon Choi, Hyue Mee Kim, Hoyoun Won, Jong-Chan Youn, Sang-Wook Kim, Wang-Soo Lee, Jun Hwan Cho, Kyung‑Taek Park, Joonhwa Hong, Da-Young Kim

**Affiliations:** 1 Red Cross College of Nursing Chung-Ang University Seoul Republic of Korea; 2 Mo-Im Kim Nursing Research Institute Yonsei University College of Nursing Yonsei University Institute for Innovation in Digital Healthcare Seoul Republic of Korea; 3 Division of Cardiology Chung-Ang University Hospital Chung-Ang University College of Medicine Seoul Republic of Korea; 4 Division of Cardiology, Department of Internal Medicine Seoul St. Mary’s Hospital Catholic Research Institute for Intractable Cardiovascular Disease, The Catholic University of Korea Seoul Republic of Korea; 5 Division of Cardiology Chung-Ang University Gwangmyeong Hospital and Department of Internal Medicine Chung-Ang University College of Medicine Gwangmyeong Republic of Korea; 6 Department of Thoracic and Cardiovascular Surgery Chung-Ang University Hospital Chung-Ang University College of Medicine Seoul Republic of Korea; 7 Graduate School of Nursing Chung-Ang University Seoul Republic of Korea

**Keywords:** behavioral change, digital interventions, family caregiver, heart failure, mHealth, patients, quasi-experimental study, quality of life, self-care, smartphone apps

## Abstract

**Background:**

A patient–family caregiver dyad approach is necessary to improve adherence to self-care behaviors by patients with heart failure (HF). However, there is a lack of mobile health (mHealth) interventions that engage both patients and their family caregivers to promote HF self-care.

**Objective:**

The purpose of this quasi-experimental study was to develop and confirm the feasibility and effectiveness of a novel mHealth intervention based on patient–family caregiver dyads for promoting adherence to self-care by patients with HF.

**Methods:**

We developed a dyadic mHealth program with 2 main features: a basic feature app and an interactive text-based chatbot. The intervention group (35 of 70 HF patient–family caregiver dyads; 50%) underwent a dyadic mHealth program for 24 weeks, while the control group (35 of 70 dyads; 50%) received usual care. Adherence to self-care behaviors, family caregivers’ contributions to self-care behaviors, and health-related quality of life were evaluated. Data were collected using self-administered questionnaires at baseline and at 1 month, 3 months, and 6 months post enrollment. The outcomes were analyzed using intention-to-treat analysis.

**Results:**

The intervention group had significantly better adherence to self-care behaviors (β=4.68, 95% CI 0.99-8.37) and family caregivers’ contributions to self-care behaviors (β=8.76, 95% CI 4.63-12.88) over 6 months compared with the control group. The 6-month follow-up health-related quality of life scores for patients (β=0.07, 95% CI 0.00-0.13) and family caregivers (β=0.08, 95% CI 0.03-0.13) were significantly greater in the intervention group than in the control group. The 1-month follow-up disease knowledge scores for patients (β=0.16, 95% CI 0.03-0.29) and family caregivers (β=0.12, 95% CI 0.00-0.25) were significantly greater in the intervention group than in the control group. The intervention also had a significant effect on mutuality at the 1-month follow-up for patients (β=0.11, 95% CI 0.00-0.21) and family caregivers (β=0.15, 95% CI 0.01-0.30). However, health literacy was significantly higher in the intervention group than in the control group only for patients at 1 month (β=0.14, 95% CI 0.04-0.25). The intervention had no significant effects on depressive symptoms, social support with patient and family caregivers, and caregiver burden with family caregivers.

**Conclusions:**

This study found that the dyadic mHealth intervention was beneficial for improving patients’ adherence to self-care behaviors and family caregivers’ contributions to self-care behaviors by providing information and motivation and improving health-related quality of life for patients with HF and family caregivers. Further studies should confirm the generalizability, feasibility, and long-term health outcomes of this intervention.

**Trial Registration:**

Clinical Research Information Service (CRIS) KCT0008786; https://tinyurl.com/3684ur4r

## Introduction

The proportion of individuals with heart failure (HF) is expected to rise with an aging population and advancements in diagnostics and therapeutics [1]. For HF, the readmission rate within 6 months is as high as 50% [1], contributing to high mortality and health care costs [2]. To improve patient outcomes, adequate self-care behaviors (eg, taking medication, limiting sodium intake, monitoring symptoms, managing exacerbations, and maintaining regular exercise) are essential for effective HF management [2,3]. Good adherence to self-care can prevent adverse events, including readmission after hospital discharge and mortality [4,5]. However, a recent systematic review of 39 empirical studies revealed that many people with HF struggle with adhering to self-care behaviors [4]. Nonadherence to HF self-care behaviors can lead to impairments in physical function caused by worsening symptoms, cognitive decline, and multimorbidity [2,3].

Family caregivers are essential for encouraging active patient involvement in their self-care [6,7]. Consequently, developing interventions that foster collaboration between patients and their family caregivers could enhance optimal HF self-care [5]. Existing evidence has shown that HF self-care interventions rarely incorporate patient preferences and family support [7]. Although family members often assist with day-to-day self-management, they have been largely excluded from most past interventions for HF self-care [7].

Self-care behaviors in chronic conditions are a dyadic coping process in which the self-care behaviors of patients with HF and their caregivers closely impact one another [5]. A report from the American Heart Association in 2020 stressed the significance of family caregiving for HF management, advocating for a dyadic approach over patient-only or family caregiver–centered models to enhance in clinical practice [7]. Collaborative efforts between patients and caregivers may strengthen patients’ self-care abilities while reducing the physical and psychological burden on family caregivers [5]. Unfortunately, a systematic review by Buck et al [8] found limited evidence supporting dyadic interventions for promoting HF self-care. The study found inconsistent HF self-care outcomes for dyadic interventions, with interventions that did not consider dyadic variables that may influence self-care, such as mutuality, limited to a few countries or not based on a theoretical framework [8].

Notably, mobile health (mHealth) interventions are increasingly used to support HF self-care behaviors regardless of time or place [9]. Evidence suggests that mHealth technologies can boost confidence with medication adherence, self-care behaviors, and lifestyle modifications [9,10]. Furthermore, a study reported that the use of mHealth technology to improve adherence to self-care behaviors resulted in reduced mortality and HF-associated readmissions [10]. However, existing mHealth apps for supporting HF self-care typically deliver unidirectional information, such as reminders or one-way communication, with short follow-up periods [9]. Some interactive mHealth apps use phone calls or SMS text messages, but these are typically confined to one-time conversations [9,11].

According to the Information-Motivation-Behavioral Skills (IMB) model [12], acquiring enough behavior-specific information can stimulate motivation and help build the skills needed to engage in targeted health behaviors. If individuals are well-informed, they will likely be motivated to initiate healthy behaviors. Health literacy refers to the process of evaluating an individual’s ability to analyze, interpret, and utilize health information, going beyond just reading and understanding materials, to make informed decisions [10]. mHealth interventions that consider users’ health literacy levels can help patients and their families access health information and make treatment decisions [11]. These interventions can also improve health literacy levels by providing access to health information anytime through a common interface, enabling the acquisition of desired health knowledge [10,11]. Therefore, previous studies have emphasized that health literacy should be considered when developing mHealth interventions to overcome barriers related to technology and enhance patients’ self-care abilities [10,11].

Previous studies regarding HF self-care have shown that mHealth interventions have primarily focused on patients with HF only or have not considered the user’s health literacy [9,13]. Therefore, mHealth interventions should be developed using a dyadic approach for better HF self-care considering health literacy. This study aimed to develop a dyadic mHealth intervention based on the IMB model to promote HF self-care behaviors and evaluate the effects on health outcomes for both patients and family caregivers at 6 months.

## Methods

### Study Design

This quasi-experimental study involved 2 steps. First, a dyadic mHealth intervention was developed for HF self-care behaviors. Second, our study was performed to demonstrate the effectiveness of dyadic mHealth interventions.

### Study Setting and Sample

We recruited individuals with HF and their family caregivers (HF dyads) from a tertiary care hospital in Seoul, Korea, between October 2023 and April 2024.

The inclusion criteria for patients with HF were as follows: (1) patients aged 18 years or older, (2) HF diagnosis by a cardiologist at least 6 months prior, and (3) HF symptoms according to New York Heart Association class II or III. For family caregivers, the inclusion criteria were as follows: (1) individuals aged 18 years or older and (2) a primary caregiver who talks with the patient about the patient’s health condition at least twice per month and regularly helps with at least one aspect of the patient’s care (eg, medications, symptom monitoring, clinic appointments).

The exclusion criteria for HF patient–family caregiver dyads were as follows: (1) inability to understand spoken or written Korean; (2) not owning or not knowing how to properly use a mobile phone compatible with the Android operating system; and (3) being diagnosed with severe mental health conditions, dementia, or complex conditions such as renal failure or terminal cancer.

The sample size calculation was conducted using G*Power 3.1.9.7. A sample size of 128 (64 in each group; 32 dyads) was calculated a priori. An effect size of 0.25 was identified, with a desired power of 0.8 (2-tailed α=.05) on all patients and their caregivers’ outcomes, assessed at 6 months post intervention. Considering an estimated attrition rate of 10%, 35 dyads were recruited for each group.

### The Dyadic mHealth Program

Our dyadic mHealth intervention for HF self-care was designed for patients with HF and family caregiver dyads. We performed an extensive literature review of dyadic research and HF self-care [2,5]. Based on our previous research on the Welcome to interactive text messaging for improving HF self-care unified supporters (WITHUS) program for patients, which was developed to promote self-care by patients with HF [11,14], we provided feedback on the health data they entered and offered information on stress management, created online peer support groups, and developed an mHealth app for family caregivers.

The dyadic mHealth program comprises a basic app with the program’s key features and a chatbot app. The basic app allowed patients and family caregivers to input their status and symptoms through button-based selections. The basic app (derived from the IMB model) for patients and their family caregivers included the following: disease and treatment information, information about a healthy diet and skills for stress management, weekly individualized goal setting, visualization of individual accomplishments based on goal achievements (blooming sunflower, ranking system), recording of self-care behaviors, writing a mood diary for managing depressive symptoms, interactive text messages using chatbots, peer groups with other patients and family caregivers, communication with research assistant nurses via voice calls, visualization and tracking of physiological (blood pressure, pulse rate, and body weight) and behavioral health data entered by the participant, personalized feedback based on inputted health data, and notifications to take medication and for hospital follow-up visits (Table 1). The patient-specific app included symptom monitoring, low-sodium diet and water restriction, blood pressure checks, and sending records of self-care behaviors to family caregivers. The family caregiver–specific app included diet, checking the health data inputted by patients, and sending notices or reminders to patients to encourage their self-care.

The chatbot app used precomposed scenarios to encourage and support dyadic HF self-care behaviors. The chatbot app used a rule-based system that provided responses based on a fixed conversation flow triggered by receiving prescripted text messages and selecting from predetermined response options. The chatbot scenarios were developed by researchers, and a cardiologist who is part of the research team validated the content by reviewing the scenarios. This structure was chosen to ensure clarity, consistency, and ease of use for older adults and caregivers, and it did not involve generative nor machine learning–based features. Messages on the chatbot app included encouragement for goal setting, information provision, and reminders. Messages were sent by the chatbot app 3 times per week for 24 weeks to patients and family caregivers. The details of the app screenshots are presented in Multimedia Appendix 1.

The intervention group was provided with the 24-week dyadic WITHUS program. After obtaining informed consent and conducting the baseline assessment, each patient–family caregiver dyad was asked to install the dyadic mHealth app on their individual mobile phones and sign in using the newly created participant accounts. The accounts of patients and their family caregivers were linked through their individual phone numbers. Subsequently, trained research assistants provided an in-person session that involved demonstrations regarding how to use the dyadic mHealth app via a 5-minute video clip and leaflet. Participants were instructed to contact the research assistant via a phone call or the messaging function in the app if they had any questions or required assistance while using the app. Two research assistants monitored and responded to questions or requests from the participants in real time via a web dashboard accessible only to the research assistant. Research assistants used a structured protocol for intervention delivery and problem-solving to maintain intervention fidelity.

Patients or family caregivers in the control group received usual care only.

**Table 1:** Components and outcome measures of dyadic mobile health (mHealth) interventions for heart failure self-care using the Information-Motivation-Behavioral Skills (IMB) model.

IMB model and components of the dyadic mHealth intervention		Study participants	Outcome measures
**Information**			
	Disease and treatment	P^a^, C^b^	Both: health literacy
	Healthy diet and stress management skills	P, C	Both: disease knowledge
**Motivation (personal)**			
	Individualized goal setting	P, C	Both: social support
	Visualization for individual accomplishments based on goal achievements	P, C	Both: mutuality
	Recording self-care behaviors	P, C	—^c^
	Writing a mood diary for managing depressive symptoms	P, C	Both: depressive symptoms
	Interactive text message using a chatbot	P, C	—
**Motivation (social)**			
	Peer group with other patients and family caregivers	P, C	—
	Communication with research assistant nurses via voice calls	P, C	—
**Behavioral skills**			
	Visualization and tracking physiological and behavioral health data	P, C	Patients: self-care behaviors; family caregivers: contributions to self-care behaviors and caregiver burden; both: health-related quality of life
	Personalized feedback based on inputted health data	P, C	Patients: self-care behaviors; family caregivers: contributions to self-care behaviors and caregiver burden; both: health-related quality of life
	Notifications to take medication and attend hospital follow-up visits	P, C	Patients: self-care behaviors; family caregivers: contributions to self-care behaviors and caregiver burden; both: health-related quality of life
	Checking patients’ inputted health data	C	Family caregivers: contributions to self-care behaviors and caregiver burden, health-related quality of life
	Sending notice or reminders to patients to encourage their self-care	C	Family caregivers: contributions to self-care behaviors and caregiver burden, health-related quality of life

^a^P: patients.

^b^C: family caregivers.

^c^Not applicable.

### Measurements

#### General Characteristics

Information on patients’ sociodemographic characteristics and clinical information were collected. In addition, information on caregivers’ general characteristics including their relationship with the patient, time spent caregiving for the patient, and hours of care provided per week were collected through a self-reported survey. The dyadic care type was assessed using a single question [15], which was asked to best describe whether the patient’s self-care was managed by the patient or family caregiver (individually oriented dyadic type) or whether the patient and family performed self-care together or complemented each other’s self-care in different areas (collaboratively oriented dyadic type). The views of the patient and family were classified as “incongruent” if they disagreed.

#### Patients’ Self-Care Behaviors

We used the European Heart Failure Self-Care Behavior Scale (EHFScBS-9) developed by Jaarsma et al [16]. Son and Won [17] evaluated the reliability and validity of the Korean version of the EHFScBS-9 with Korean patients with HF. The scale consists of 9 questions. Each item is given a score from 1 (not at all) to 5 (very much so). The overall score ranges from 9 to 45. Higher scores indicate greater levels of self-care behavior. For this study, the Cronbach α was 0.76 to 0.88.

#### Family Caregivers’ Contributions to Self-Care Behaviors

To measure the self-care behaviors of family caregivers of patients with HF, we used the EHFScBS-Caregiver version (EHFScBS-C) developed by Durante et al [18]. The EHFScBS-C was translated to Korean using forward and backward translation and linguistically validated by the authors. This 9-item tool measures the same self-care behaviors as the patient version, with each item asking the extent to which the family is involved in the patient’s care. The total score range is 0 to 45. Higher scores indicate family caregivers’ active involvement with helping with adherence to self-care behaviors by patients with HF. The Cronbach α was 0.82 to 0.91 in this study.

#### Health-Related Quality of Life

The Korean version of the 5-level EuroQol 5-dimensional questionnaire was adopted to evaluate health-related quality of life (HRQOL) [19]. This scale includes 5 domains: usual activities, mobility, self-care, pain or discomfort, and anxiety or depression. Each domain has 5 response levels. According to this tool, the closer the index is to 1, the more satisfied a person is with their HRQOL.

#### Health Literacy

We used the Brief Health Literacy Screener to evaluate health literacy [20]. It consists of 3 items with a 5-point scale (0-4). The total score range is 0 to 12, with greater scores indicating higher health literacy. The reliability for the Korean version was a Cronbach α value of 0.82 [21]. The Cronbach α was 0.91 to 0.96 for patients and 0.85 to 0.94 for family caregivers in this study.

#### Disease Knowledge

We used the Korean version of the Patient Knowledge Questionnaire developed by Son and Song [21]. This instrument was initially developed by Lainscak and Keber [22]. The instrument comprises 10 questions. Each question can be answered with a “yes” or “no” response, with 1 point given for each correct answer. The total score ranges from 0 to 10. Higher scores indicate greater HF knowledge. When translating the Korean version of the Patient Knowledge Questionnaire, reliability according to the Kuder-Richardson 20 was 0.80 [21]. In this study, reliability calculated through the Kuder-Richardson 20 was 0.58 to 0.83 for patients and 0.62 to 0.84 for family caregivers.

#### Depressive Symptoms

We used the Korean version of the Patient Health Questionnaire-9 [23,24]. This instrument comprises 9 questions measured on a 4-point scale (0 for “never” to 3 for “almost every day”). Higher scores indicate more severe depressive symptoms. The Cronbach α for patients and family caregivers in this study was 0.63 to 0.82 and 0.71 to 0.89, respectively.

#### Social Support

We adopted the Korean version of the ENRICHD Social Support Instrument [25]. This instrument was developed by Mitchell et al [26]. This tool includes 7 items with a 5-point Likert scale. Higher scores indicate greater social support; the overall score ranges from 8 to 34. The reliability of the tool at the time of development was a Cronbach α of 0.86 [26]. In the study by Jo et al [25] with Korean patients with HF, the Cronbach α was 0.96. In this study, the Cronbach α was 0.86 to 0.95 for patients and 0.84 to 0.90 for family caregivers.

#### Mutuality

Mutuality is defined as “the quality of a positive association between the patient and caregiver” [27]. We used a mutuality scale [27], which was translated and backtranslated into Korean by our team. The content validity was confirmed. Each item was assessed on a 5-point Likert scale (0=“not at all” to 4=“a great deal”). The mean score, calculated by averaging all 15 items, ranges from 0 to 4, with higher scores indicating better overall mutuality. The Cronbach α at the time of development was 0.91 for patients and 0.94 for caregivers [27]. In this study, the Cronbach α was 0.97 to 0.98 for patients and 0.96 to 0.98 for family caregivers.

#### Caregiver Burden

We used the Heart Failure Caregiver Questionnaire developed by Strömberg et al [28]. The tool was translated and backtranslated into Korean by our research team, and its content validity was confirmed. The tool comprises 21 questions, and each item is given a score from 1 (not at all) to 5 (a lot). Overall scores range from 0 to 100. Higher scores indicate a higher subjective burden associated with caregiving. The Cronbach α was 0.86 to 0.95 in this study.

#### Assessment of the Usability of the Dyadic mHealth Intervention

To test the usability of the app, we used the Mobile Application Rating Scale user version developed by Stoyanov et al [29]. This tool served as a quality assessment for health-related apps in the experimental group during the 6-month follow-up. The overall app quality score comprises 16 items divided into 4 subscales (ie, engagement, functionality, aesthetics, and information). Each item was assessed on a 5-point scale ranging from 1 to 5, with a higher rating reflecting better app quality. The internal consistency reported by the development study was a Cronbach α of 0.90 [29]. In this study, the Cronbach α was 0.85 for patients and 0.82 for family caregivers. Furthermore, we monitored the time patients and family caregivers spent on each feature of the app each week through the web dashboard. We also tracked the number of clicks to send patients’ inputted health data to the family caregivers’ app or that of family caregivers to identify patients’ input health data.

### Study Procedure

We recruited the experimental group after recruiting the control group to prevent contamination. Cardiologists who were members of the research team conducted the initial screening and provided a list of potential patients. Two research assistants screened the patients and contacted their family caregivers, either in person or by phone, to assess their eligibility.

This study used a single-blind design: participants (patients and their family caregivers) and cardiologists were blinded to the group assignments, whereas the research assistants responsible for intervention delivery and data collection were not. We collected data at 4 time points: (1) an in-person baseline assessment conducted immediately after enrollment and before the intervention began and (2) three follow-ups at 1, 3, and 6 months postenrollment, completed either in person or via telephone, based on the participant’s preference. Patients with HF and family caregiver dyads completed surveys independently in separate rooms. Patients’ clinical information was obtained from electronic medical records.

### Ethical Considerations

For this study, ethical approval was obtained from the Institutional Review Board of Chung-Ang University (number 1041078-20230520-HR-140). All participants provided written informed consent before data collection and the intervention. Participant data were anonymized and deidentified. Participants were provided with a blood pressure monitor and salinity meter as compensation for their participation.

### Data Analysis

Statistical analyses were undertaken using SPSS Statistics for Windows (version 28.0; IBM Corp). Using the Shapiro-Wilk test, the normality of continuous variables was evaluated. Participant characteristics were reported using descriptive statistics. To evaluate homogeneity of baseline measures between the groups, we conducted independent-samples *t* tests, Mann-Whitney *U* tests, chi-square tests, and Fisher exact tests. The effectiveness of the intervention was tested using generalized estimating equations (GEEs). The analyses used a first-order autoregressive structure assuming that the responses at each time point were influenced by adjacent points. An intention-to-treat analysis was conducted to mitigate the impact of participant dropout. Missing data were not imputed because the GEE assumes random missing values, and baseline data were available for all participants [30]. A *P* value <.05 was considered statistically significant.

## Results

### Sample Characteristics

A total of 129 patients with HF were enrolled. Of these, 37 were excluded because of the absence of a caregiver (n=5), of the lack of mobile phone ownership (n=2), or they declined to participate (n=30). After caregiver involvement was assessed, 92 dyads were further screened. Of these, 22 were excluded because a caregiver had a history of psychiatric illness (n=1) or declined to participate (n=21). A total of 70 dyads were included (intervention group: 35 dyads; control group: 35 dyads).

Retention rates varied across follow-up assessments. In the intervention group, attrition occurred because of patients declining to participate (3 dyads), hospital transfer (1 dyad), and loss to follow-up (1 dyad), with 30 dyads remaining at the 6-month follow-up. In the control group, dropouts were due to declining participation (4 dyads), loss to follow-up (1 dyad), and patient death (1 dyad), leaving 29 dyads at 6 months. Despite attrition, an intention-to-treat analysis was conducted, including all initially enrolled dyads (35 dyads per group; see Multimedia Appendix 2).

Table 2 presents the baseline characteristics of the 70 dyads. The mean age of patients was 70.67 (SD 11.48) years, while the mean age of family caregivers was 59.34 (SD 14.38) years. The majority of patients were men (n=45, 64%), whereas most family caregivers were women (n=53, 76%). Family caregivers were primarily spouses (n=44, 63%), and a high number of caregivers lived with the patient (n=58, 83%). Regarding the dyadic care type, only 29% (n=20) of the dyads collaboratively engaged in self-care behaviors. The average scores for self-care behavior were 28.06 (SD 6.69) for patients and 27.73 (SD 9.04) for family caregivers. Regarding HRQOL, patients averaged a score of 0.90 (SD 0.11), and family caregivers averaged a score of 0.92 (SD 0.10). There was no significant difference in baseline characteristics between the 2 groups.

**Table 2:** Homogeneity of baseline characteristics between the intervention and control groups (70 dyads).

Categories			Patients												Family caregivers											
			Intervention group (n=35)		Control group (n=35)		Statistic (*df*)			*P* value			Intervention group (n=35)				Control group (n=35)			Statistic (*df*)				*P* value		
Age (years), mean (SD)			69.49 (1.72)		71.86 (2.15)		–0.86 (68)^a^			.39			62.03 (1.84)				56.66 (2.86)			1.58 (68)^a^				.12		
**Gender, n (%)**								0.06 (1)^b^			.80										3.8 (1)^b^				.051	
	Men	23 (66)		22 (63)								5 (14)				12 (34)										
	Women	12 (34)		13 (37)								30 (86)				23 (66)										
**Education level, n (%)**								0.08 (2)^b^			.96										0.2 (2)^b^				.88	
	Below middle school	12 (34)		13 (37)								7 (20)				8 (23)										
	High school	11 (31)		11 (31)								15 (43)				13 (37)										
	Above college	12 (34)		11 (31)								13 (37)				14 (40)										
**Employed, n (%)**								0.2 (1)^b^			.63										0.06 (1)^b^				.81	
	Yes	15 (43)		13 (37)								19 (54)				18 (51)										
	No	20 (57)		22 (63)								16 (46)				17 (49)										
**Monthly income (₩^b^), n (%)**								3.9 (1)^c^			.048										0.01 (1)^c^				≥.99	
	<2,000,000	18 (51)		26 (74)								20 (57)				20 (57)										
	≥2,000,000	17 (49)		9 (26)								15 (43)				15 (43)										
**Time since HF^d^ diagnosis (years), n (%)**								1.0 (2)^c^			.60															
	<1	6 (17)		9 (26)								—^e^				—			—							
	1-3	6 (17)		4 (11)								—				—			—							
	≥3	23 (66)		22 (63)								—				—			—							
**NYHA^f^ class**								0.2 (1)^c^			.63															
	Ⅱ	16 (46)		18 (51)								—				—			—							
	Ⅲ	19 (54)		17 (49)								—				—			—							
**Relationship with patient, n (%)**																						2.19^g^				.43
	Spouse	—		—		—			—			25 (71)				19 (54)										
	Child	—		—		—			—			7 (20)				11 (31)										
	Siblings and parents	—		—		—			—			3 (9)				5 (14)										
**Living with patient, n (%)**																						1.6 (1)^c^				.21
	Yes	—		—		—			—			31 (89)				27 (77)										
	No	—		—		—			—			4 (11)				8 (23)										
**Duration of caregiving (years), n (%)**																						2.3 (2)^c^				.32
	<1	—		—		—			—			8 (23)				10 (29)										
	1-3	—		—		—			—			5 (14)				9 (26)										
	≥3	—		—		—			—			22 (63)				16 (46)										
**Time spent in caregiving (hours/week), n (%)**																						7.6 (3)^c^				.05
	≤7	—		—		—			—			13 (37)				4 (11)										
	8-14	—		—		—			—			7 (20)				13 (37)										
	15-26	—		—		—			—			6 (17)				10 (29)										
	≥27	—		—		—			—			9 (26)				8 (23)										
**Dyadic care type, n (%)**								3.3 (2)^c^			.19															
	Individually oriented	16 (46)		17 (49)								—				—			—							
	Collaboratively oriented	13 (37)		7 (20)								—				—			—							
	Incongruent	6 (17)		11 (31)								—				—			—							
**Outcome variables, mean (SD)**																										
	Health literacy	8.37 (3.06)		8.06 (2.96)		0.88^h^			.38			9.17 (2.42)				9.09 (2.31)			0.23^h^				.82			
	Disease knowledge	6.06 (2.36)		5.20 (2.15)		1.59 (68)^a^			.12			6.80 (2.18)				5.86 (2.39)			1.62 (68)^a^				.11			
	Depressive symptoms	3.43 (3.56)		3.34 (3.47)		0.27^h^			.82			3.43 (3.56)				2.00 (2.66)			1.86^h^				.06			
	Social support	29.00 (5.17)		28.91 (5.66)		0.16^h^			.87			30.20 (4.40)				30.20 (4.46)			–0.01^h^				.99			
	Mutuality	2.76 (0.87)		2.55 (0.99)		0.83^h^			.41			2.55 (0.79)				2.27 (0.97)			1.35^h^				.18			
	Self-care behaviors	27.26 (6.81)		28.86 (6.55)		–1.00 (68)^a^			.32			—				—			—				—			
	Contribution to self-care behaviors	—		—		—			—			26.46 (10.51)				29.00 (7.21)			–1.18 (68)^a^				.24			
	Caregiver burden	—		—		—			—			18.32 (15.34)				13.54 (11.70)			0.97^h^				.33			
	Health-related quality of life	0.90 (0.11)		0.90 (0.10)		0.34^h^			.74			0.89 (0.11)				0.94 (0.09)			–1.80^h^				.07			

^a^*t* test.

^b^A currency exchange rate of ₩1300=US $1 is applicable.

^c^Chi-square test.

^d^HF: heart failure.

^e^Not applicable.

^f^NYHA: New York Heart Association.

^g^Fisher exact test.

^h^Mann-Whitney *U* test.

### Effect of a Dyadic mHealth Intervention on Outcome Variables for Patients With HF

The GEE analysis revealed that participants in the intervention group had a significantly greater increase in patients’ self-care behaviors from baseline at all follow-up time points, compared with the control group: 1 month (β=5.29, 95% CI 1.18-8.76; *P*=.003), 3 months (β=4.77, 95% CI 1.26-8.29; *P*=.008), and 6 months (β=4.68, 95% CI 0.99-8.37; *P*=.01; Table 3). The intervention group had significantly greater improvements in patients’ HRQOL scores at the 6-month follow-up (β=0.07, 95% CI 0.00-0.13; *P*=.049) than the control group. However, these differences were not significant at the 1-month (β=0.02, 95% CI –0.01 to 0.04; *P*=.15) and 3-month (β=0.05, 95% CI –0.01 to 0.10; *P*=.10) follow-ups.

**Table 3:** Effectiveness of dyadic mobile health (mHealth) interventions among patients with heart failure.

Outcome variables and measurement time point		Intervention group, mean (SD)	Control group, mean (SD)	Group effect^a^					Time effect^b^				Group × time effect^c^		
				β (95% CI)		*P* value		β (95% CI)		*P* value		β (95% CI)		*P* value	
**Health literacy**					0.03 (–0.12 to 0.18)		.66								
	Baseline	8.37 (3.06)	8.06 (2.96)					Reference		—^d^		Reference		—	
	1-month follow-up	9.51 (2.88)	7.80 (2.47)					–0.03 (–0.10 to 0.04)		.42		0.14 (0.04 to 0.25)		.008	
	3-month follow-up	8.91 (2.90)	9.24 (3.87)					0.12 (0.02 to 0.23)		.02		–0.08 (–0.22 to 0.06)		.26	
	6-month follow-up	8.40 (2.92)	8.74 (3.16)					0.04 (–0.06 to 0.15)		.43		–0.05 (–0.19 to 0.08)		.45	
**Disease knowledge**					0.13 (–0.03 to 0.29)		.11								
	Baseline	6.06 (2.36)	5.20 (2.15)					Reference		—		Reference		—	
	1-month follow-up	7.46 (2.74)	5.31 (2.23)					0.02 (–0.08 to 0.11)		.70		0.16 (0.03 to 0.29)		.01	
	3-month follow-up	8.30 (2.60)	6.76 (2.72)					0.22 (0.10 to 0.35)		.001		0.04 (–0.12 to 0.19)		.62	
	6-month follow-up	8.57 (2.46)	7.23 (2.73)					0.27 (0.14 to 0.40)		<.001		0.0 (–0.15 to 0.15)		≥.99	
**Depressive symptoms**					0.02 (–0.35 to 0.39)		.92								
	Baseline	3.43 (3.56)	3.34 (3.47)					Reference		—		Reference		—	
	1-month follow-up	1.29 (1.69)	1.89 (2.01)					–0.41 (–0.64 to –0.18)		.001		–0.25 (–0.60 to 0.10)		.15	
	3-month follow-up	1.79 (2.65)	2.56 (4.03)					–0.21 (–0.58 to 0.16)		.27		–0.26 (–0.8 to 0.28)		.35	
	6-month follow-up	1.43 (1.85)	2.35 (3.25)					–0.26 (–0.62 to 0.09)		.15		–0.30 (–0.78 to 0.17)		.21	
**Social support**					0.00 (–0.08 to 0.09)		.95								
	Baseline	29.00 (5.17)	28.91 (5.66)					Reference		—		Reference		—	
	1-month follow-up	30.54 (4.75)	29.97 (5.60)					0.04 (–0.02 to 0.09)		.18		0.02 (–0.06 to 0.09)		.68	
	3-month follow-up	30.24 (5.95)	28.21 (7.32)					–0.02 (–0.11 to 0.07)		.63		0.07 (–0.04 to 0.17)		.21	
	6-month follow-up	29.20 (5.06)	28.32 (6.26)					–0.03 (–0.12 to 0.05)		.47		0.03 (–0.08 to 0.14)		.54	
**Mutuality**					0.08 (–0.09 to 0.24)		.35								
	Baseline	2.76 (0.87)	2.55 (0.99)					Reference		—		Reference		—	
	1-month follow-up	3.10 (0.77)	2.57 (0.92)					0.01 (–0.08 to 0.09)		.86		0.11 (0.00 to 0.21)		.04	
	3-month follow-up	3.26 (0.80)	2.53 (0.86)					–0.01 (–0.13 to 0.11)		.83		0.16 (0.02 to 0.31)		.03	
	6-month follow-up	3.08 (0.78)	2.56 (0.86)					0.00 (–0.11 to 0.11)		.98		0.09 (–0.05 to 0.22)		.22	
**Self-care behaviors**					–1.60 (–4.69 to 1.49)		.31								
	Baseline	27.26 (6.81)	28.86 (6.55)					Reference		—		Reference		—	
	1-month follow-up	32.89 (6.97)	29.20 (6.08)					0.34 (–1.75 to 2.43)		.75		5.29 (1.81 to 8.76)		.003	
	3-month follow-up	35.85 (6.61)	32.71 (7.90)					3.82 (2.07 to 5.57)		<.001		4.77 (1.26 to 8.29)		.008	
	6-month follow-up	34.40 (6.11)	31.42 (5.90)					2.31 (0.23 to 4.39)		.03		4.68 (0.99 to 8.37)		.01	
**Health-related quality of life**					0.00 (–0.06 to 0.05)		.87								
	Baseline	0.90 (0.11)	0.90 (0.10)					Reference		—		Reference		—	
	1-month follow-up	0.90 (0.11)	0.89 (0.12)					–0.02 (–0.04 to 0.01)		.15		0.02 (–0.01 to 0.04)		.15	
	3-month follow-up	0.93 (0.12)	0.88 (0.14)					–0.03 (–0.07 to 0.02)		.24		0.05 (–0.01 to 0.10)		.10	
	6-month follow-up	0.94 (0.08)	0.88 (0.15)					–0.03 (–0.07 to 0.02)		.31		0.07 (0.00 to 0.13)		.049	

^a^Reference: control group.

^b^Reference: baseline time.

^c^Reference: control group × baseline time.

^d^Not applicable.

For health literacy and disease knowledge, the intervention group had a significantly greater increase from baseline than the control group, but only at the 1-month follow-up (health literacy: β=0.14, 95% CI 0.04-0.25; *P*=.008; disease knowledge: β=0.16, 95% CI 0.03-0.29; *P*=.01). Similarly, the intervention group had a significantly greater increase in mutuality from baseline to 1 month (β=0.11, 95% CI 0.00-0.21; *P*=.04) and 3 months (β=0.16, 95% CI 0.02-0.31; *P*=.03). In contrast, the 2 groups were not significantly different in terms of symptoms of depression nor social support.

### Effect of the Dyadic mHealth Intervention on Outcome Variables for Family Caregivers

The effects of the intervention among family caregivers are presented in Table 4. Individuals in the intervention group had significantly greater improvements in their contribution to self-care behaviors from baseline to 1 month (β=3.53, 95% CI 0.23-6.84; *P*=.04), 3 months (β=9.35, 95% CI 4.83-13.88; *P*<.001), and 6 months (β=8.76, 95% CI 4.63-12.88; *P*<.001) compared with the control group. In addition, for HRQOL, the intervention group had a significantly greater improvement than the control group from baseline to 6 months (β=0.08, 95% CI 0.03-0.13; *P*=.001).

**Table 4:** Effectiveness of dyadic mobile health (mHealth) interventions for heart failure self-care by family caregivers.

Outcome variables	Time	Intervention group, mean (SD)	Control group, mean (SD)	Group effect^a^				Time effect^b^				Group × time effect^c^			
				β (95% CI)		*P* value		β (95% CI)		*P* value		β (95% CI)		*P* value	
**Health literacy**					0.01 (–0.11 to 0.13)		.88								
	Baseline	9.17 (2.42)	9.09 (2.31)					Reference		—^d^		Reference		—	
	1-month follow-up	9.88 (2.11)	9.00 (1.90)					0.00 (–0.07 to 0.07)		.97		0.08 (–0.01 to 0.17)		.08	
	3-month follow-up	10.18 (2.44)	11.10 (1.66)					0.20 (0.13 to 0.27)		<.001		–0.10 (–0.21 to 0.01)		.09	
	6-month follow-up	9.93 (1.71)	9.83 (2.39)					0.08 (0.00 to 0.16)		.05		–0.02 (–0.14 to 0.09)		.70	
**Disease knowledge**					0.15 (–0.02 to 0.32)		.08								
	Baseline	6.80 (2.18)	5.86 (2.39)					Reference		—		Reference		—	
	1-month follow-up	7.29 (2.42)	5.41 (2.34)					–0.05 (–0.16 to 0.06)		.40		0.12 (0.00 to 0.25)		.048	
	3-month follow-up	7.91 (2.04)	7.42 (1.88)					0.25 (0.12 to 0.38)		<.001		–0.11 (–0.26 to 0.05)		.19	
	6-month follow-up	8.34 (1.86)	7.17 (1.95)					0.21 (0.08 to 0.34)		.002		–0.04 (–0.20 to 0.13)		.68	
**Depressive symptoms**					0.39 (0.00 to 0.78)		.51								
	Baseline	3.43 (3.56)	2.00 (2.66)					Reference		—		Reference		—	
	1-month follow-up	2.21 (3.19)	1.13 (1.58)					–0.36 (–0.59 to –0.13)		.002		0.03 (–0.31 to 0.38)		.86	
	3-month follow-up	1.45 (2.09)	0.84 (1.27)					–0.49 (–0.81 to –0.17)		.003		–0.11 (–0.56 to 0.34)		.63	
	6-month follow-up	1.72 (2.42)	1.28 (3.05)					–0.27 (–0.79 to 0.25)		.32		–0.21 (–0.85 to 0.43)		.52	
**Social support**					0.00 (–0.07 to 0.07)		≥.99								
	Baseline	30.20 (4.40)	30.20 (4.46)					Reference		—		Reference		—	
	1-month follow-up	29.94 (5.09)	30.53 (4.61)					0.01 (–0.04 to 0.07)		.64		–0.02 (–0.11 to 0.06)		.59	
	3-month follow-up	29.76 (4.24)	29.03 (4.44)					–0.04 (–0.11 to 0.03)		.24		0.03 (–0.06 to 0.12)		.55	
	6-month follow-up	29.93 (3.69)	28.31 (4.74)					–0.06 (–0.12 to –0.01)		.02		0.06 (–0.02 to 0.13)		.14	
**Mutuality**					0.12 (–0.06 to 0.29)		.18								
	Baseline	2.55 (0.79)	2.27 (0.97)					Reference		—		Reference		—	
	1-month follow-up	2.86 (0.91)	2.19 (0.59)					–0.04 (–0.16 to 0.08)		.52		0.15 (0.01 to 0.30)		.04	
	3-month follow-up	3.15 (0.76)	2.92 (0.67)					0.25 (0.11 to 0.38)		<.001		–0.03 (–0.20 to 0.14)		.72	
	6-month follow-up	3.08 (0.71)	2.70 (0.68)					0.17 (0.06 to 0.29)		.003		0.01 (–0.15 to 0.17)		.87	
**Contribution to self-care behaviors**					–2.54 (–6.7 to 1.62)		.23								
	Baseline	26.46 (10.51)	29.00 (7.21)					Reference		—		Reference		—	
	1-month follow-up	30.21 (8.47)	29.63 (6.16)					0.32 (–1.68 to 2.31)		.76		3.53 (0.23 to 6.84)		.04	
	3-month follow-up	32.03 (7.19)	25.55 (9.87)					–3.59 –7.15 to –0.03)		.048		9.35 (4.83, 13.88)		<.001	
	6-month follow-up	30.24 (5.94)	24.45 (6.66)					–4.45 (–7.03 to –1.86)		.001		8.76 (4.63 to 12.88)		<.001	
**Caregiver burden**					0.28 (–0.08 to 0.65)		.13								
	Baseline	18.32 (15.34)	13.54 (11.70)					Reference		—		Reference		—	
	1-month follow-up	11.10 (12.29)	6.09 (3.14)					–0.72 (–0.96 to –0.47)		<.001		0.24 (–0.12 to 0.61)		.19	
	3-month follow-up	11.29 (17.73)	6.57 (10.69)					–0.66 (–1.22 to –0.10)		.02		0.19 (–0.55 to 0.94)		.61	
	6-month follow-up	11.72 (14.90)	10.86 (14.01)					–0.20 (–0.60 to 0.21)		.35		–0.18 (–0.75 to 0.40)		.55	
**Health-related quality of life**					–0.05 (–0.10 to 0.00)		.07								
	Baseline	0.89 (0.11)	0.94 (0.09)					Reference		—		Reference		—	
	1-month follow-up	0.94 (0.08)	0.95 (0.09)					0.02 (–0.01 to 0.04)		.22		0.03 (–0.01 to 0.08)		.18	
	3-month follow-up	0.94 (0.08)	0.96 (0.10)					0.02 (–0.02 to 0.06)		.30		0.04 (–0.02 to 0.10)		.25	
	6-month follow-up	0.97 (0.07)	0.93 (0.09)					0.00 (–0.04 to 0.03)		.80		0.08 (0.03 to 0.13)		.001	

^a^Reference: control group.

^b^Reference: baseline time.

^c^Reference: control group × baseline time.

^d^Not applicable.

For disease knowledge, participants in the intervention group had significantly greater improvements than the control group from baseline but only at 1 month (β=0.12, 95% CI 0.00-0.25; *P*=.048). Similarly, the intervention group had a significantly greater increase in mutuality than the control group from baseline to 1 month (β=0.15, 95% CI 0.01-0.30; *P*=.04). However, no significant differences were found between the intervention and control groups in health literacy, symptoms of depression, social support, nor caregiver burden.

### Usability of the Intervention

The usability evaluation of the dyadic WITHUS program is presented in Multimedia Appendix 3. The median scores for overall app quality from patients and family caregivers were 2.6 (IQR 2.5-2.7) and 3.9 (IQR 3.9-4.0), respectively. Among the app quality subscales, patients rated functionality highest (median 4.4, IQR 4.3-4.5), whereas family caregivers rated information the highest (median 4.3, IQR 4.3-4.3). For the subjective app quality subscales, the median overall rating scores were 4.0 (IQR 3.3-4.0) for patients and 3.0 (IQR 3.0-4.0) for caregivers. On the perceived impact subscale, patients (median 4.5, IQR 4.0-5.0) and family caregivers (median 4.0, IQR 4.0-4.3) rated behavioral change as having the highest impact.

On average, patients used the app for 79.05 (SD 10.23) minutes per week, while family caregivers used it for 69.50 (SD 10.85) minutes per week. Patients and family caregivers spent the most time on blood pressure monitoring, followed by stress management (Multimedia Appendix 4). Interaction data (Multimedia Appendix 4) showed that patients most frequently shared medication and blood pressure records with their family caregivers. Conversely, family caregivers most often checked the patients’ salt and water intake records, followed by symptom monitoring and stress management.

## Discussion

### Principal Findings

This is one of the first studies to estimate the feasibility and effectiveness of an IMB model–based HF patient–family caregiver dyad mHealth intervention by emphasizing patients and family caregivers’ engagement in HF self-care. Our novel dyadic mHealth intervention resulted in significant enhancements in self-care behaviors and HRQOL of both patients with HF and their family caregivers. Furthermore, the intervention significantly improved patients’ health literacy, disease knowledge, and mutuality, as well as family caregivers’ disease knowledge and mutuality. These findings underscore the significance of dyadic engagement in fostering self-care at home for patients with HF.

Our dyadic mHealth intervention (WITHUS) program demonstrated a significant impact on patients’ self-care behaviors and improvements in HRQOL. Our results are consistent with those of systematic reviews on a patient-oriented mHealth intervention [31] and dyadic intervention [32]. In this study, a dyadic approach resulted in smaller increases in patients’ self-care behavior performance scores over 6 months. Considering patients with HF are typically older and may face aging-related physical and cognitive limitations, family engagement is pivotal for better self-care. Therefore, an mHealth intervention specifically designed for older patients with HF may be more suitable for improving their self-care levels and subsequent health outcomes. Further research should examine the impact of diverse mHealth intervention designs on older patients with HF to ensure that dyadic mHealth interventions are tailored to their needs.

A significant effect of the program was observed for improving family caregivers’ contributions to self-care behaviors, consistent with findings from a motivational interview intervention for HF patients [33]. However, this study found a significant difference between the control and experimental groups, whereas Locatelli et al [33] did not report such an effect. This discrepancy may be due to differences in the intervention and evaluation methods. Our study used an mHealth intervention with personalized continuous feedback, whereas Locatelli et al [33] used a motivational interviewing technique that relied on face-to-face interactions. mHealth interventions may be more effective at enhancing caregivers’ self-care behaviors because they eliminate the time and location constraints typical of face-to-face interventions, allowing for more frequent participation. Furthermore, the app enabled the monitoring of patients’ self-care, which can positively influence caregivers’ contributions to self-care behaviors.

Our dyadic mHealth intervention demonstrated a positive effect on family caregivers’ HRQOL at the 6-month follow-up. This result was similar to that of a study of a psychoeducational intervention for cancer patient–family caregiver dyads, which also reported improvements in HRQOL post intervention [34]. Patient-caregiver dyad interventions may improve health outcomes for both, maximize health care efficiency, reduce resource consumption, and yield long-term cost savings [8]. Specifically, mHealth interventions may reduce the barriers of time and space found in traditional dyadic interventions, offering a more accessible approach. Future research should assess the cost-effectiveness of mHealth interventions for patient–family caregiver dyads within the health care system to determine their potential for conserving resources and reducing long-term health care costs.

Regarding health literacy and disease knowledge, the intervention significantly improved these variables for patients and family caregivers but only at the 1-month follow-up. This is consistent with the rapid increase in health literacy and disease knowledge scores observed in a prior study [32]. When it comes to disease knowledge, our study revealed no significant differences between the 2 groups at both 3 months and 6 months post intervention among family caregivers. This may be due to the testing effect, in which the control group participants improved their knowledge through repeated surveys. We also found no statistically significant difference in health literacy among family caregivers. This could be explained by caregivers’ initial health literacy (mean score 9.13 at baseline). According to a systematic review on mHealth and health literacy [10], mHealth interventions tend to improve health literacy most significantly among individuals with low educational or health literacy levels.

Our dyadic mHealth intervention did not significantly affect social support for patients with HF or their family caregivers, which contrasts with the findings of a meta-analysis that reported improvements in social support for caregivers of patients with HF through dyad-targeted interventions [32]. However, the program significantly improved the mutuality between patients with HF and their family caregivers, aligning with a study that found that a psychoeducational intervention for patients with stroke and their caregivers positively improved their dyadic relationships [35]. The dyadic WITHUS program aims to enhance patients’ self-care and increase family caregiver involvement, which is likely to improve the mutuality between them. Since the dyadic WITHUS program primarily aims to enhance patients’ self-care and increase family caregiver involvement, improvement in the mutuality was expected, while broader social support may be beyond its scope. However, it remains unclear whether mutuality had a significant impact on self-care behaviors by patients with HF, family caregivers’ contributions to self-care behaviors, and HRQOL for patients with HF and family caregivers. Therefore, we suggest that future research should examine whether mutuality between patients with HF and family caregivers affects self-care behaviors and HRQOL.

### Limitations

First, this study adopted a quasi-experimental design, which has inherent limitations. This design can carry the risk of selection and reporting biases. In addition, research assistants delivering interventions and collecting data were not blinded, which carries the risk of observer bias and potential performance bias. Second, respondent fatigue can be induced by measuring multiple variables at multiple points in time, which can lead to nonresponse bias, potentially lower quality data, and dropouts. Therefore, future studies with randomized controlled trial designs are needed to reevaluate the effects of the intervention on certain variables for patients and family caregivers. Third, more than 50% of the caregivers in our sample had been providing care for a patient with HF for more than 3 years, whereas more than one-half spent less than 20 hours per week on caregiving, an expected duration for individuals with chronic illnesses. Consequently, these caregivers likely developed established routines and became accustomed to specific caregiving tasks, which may have reduced their responsiveness to changes. This, in turn, could have limited the impact of the intervention on behavioral patterns. Therefore, more clinical trials are required to confirm the effectiveness of our dyadic mHealth intervention in HF patient-caregiver dyads. Fourth, as social support extends beyond the patient-caregiver relationship to broader social networks, the program may be limited. Online interactions with peer groups through the app primarily involved information sharing, which may not provide the sustained emotional social support found in traditional interventions. To address this limitation, future research should explore hybrid approaches that combine online and offline interventions, such as community-based support groups and in-person meetings, to foster stronger social support networks. Finally, our dyadic mHealth intervention did not demonstrate long-term effectiveness. Future research should explore strategies to maintain engagement beyond the intervention period to ensure long-term benefits with more diverse demographics and varying levels of health literacy.

### Conclusion

The dyadic mHealth intervention for HF self-care improved patients’ adherence to self-care behaviors, family caregivers’ contributions to patients’ self-care behaviors, and HRQOL of the HF dyads. In addition, our intervention had a beneficial effect on improving disease knowledge and mutuality of HF dyads. Future research should assess the long-term effectiveness of a dyadic mHealth intervention with larger and diverse populations. More studies are required to explore the psychosocial outcomes of integrating online and offline dyadic mHealth interventions for better HF self-care.

## Appendix

Multimedia Appendix 1 Patient and family caregiver interactions within dyadic WITHUS.

Multimedia Appendix 2 Flow chart of this study.

Multimedia Appendix 3 User version of the Mobile Application Rating Scale (uMARS) scores after a 24-week intervention.

Multimedia Appendix 4 Usage time and number of clicks by features in dyadic WITHUS.

## Abbreviations

EHFScBS-9: European Heart Failure Self-Care Behavior Scale
EHFScBS-C: European Heart Failure Self-Care Behavior Scale-Caregiver version
GEE: generalized estimating equation
HF: heart failure
HRQOL: health-related quality of life
IMB: Information-Motivation-Behavioral Skills
mHealth: mobile health
WITHUS: Welcome to interactive text messaging for improving HF self-care unified supporters

## Data Availability

The data that support the findings of this study are available from the corresponding author upon reasonable request.
