# Supplementary material for: Development and Evaluation of a Patient–Family Caregiver Dyad mHealth Intervention for Heart Failure Self-Care: Quasi-Experimental Study
Source: J Med Internet Res. 2025 Jun 16;27:e74922. doi: 10.2196/74922 (PMC12209723; doi:10.2196/74922)
Supplement: Multimedia Appendix 1 [file jmir_v27i1e74922_app1.doc]

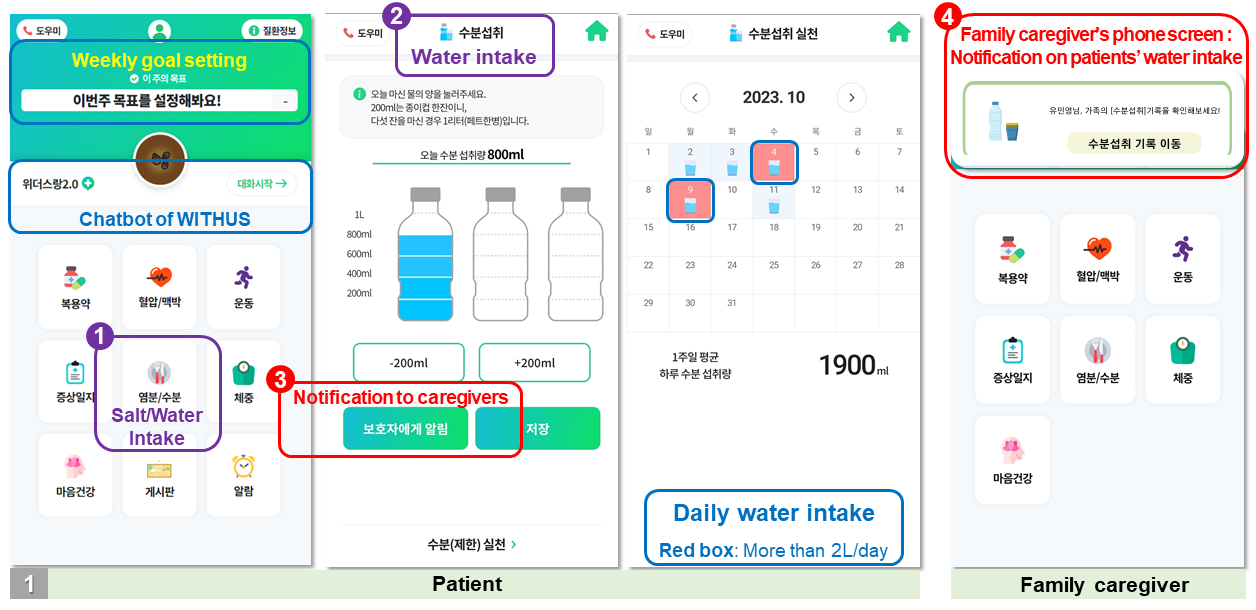


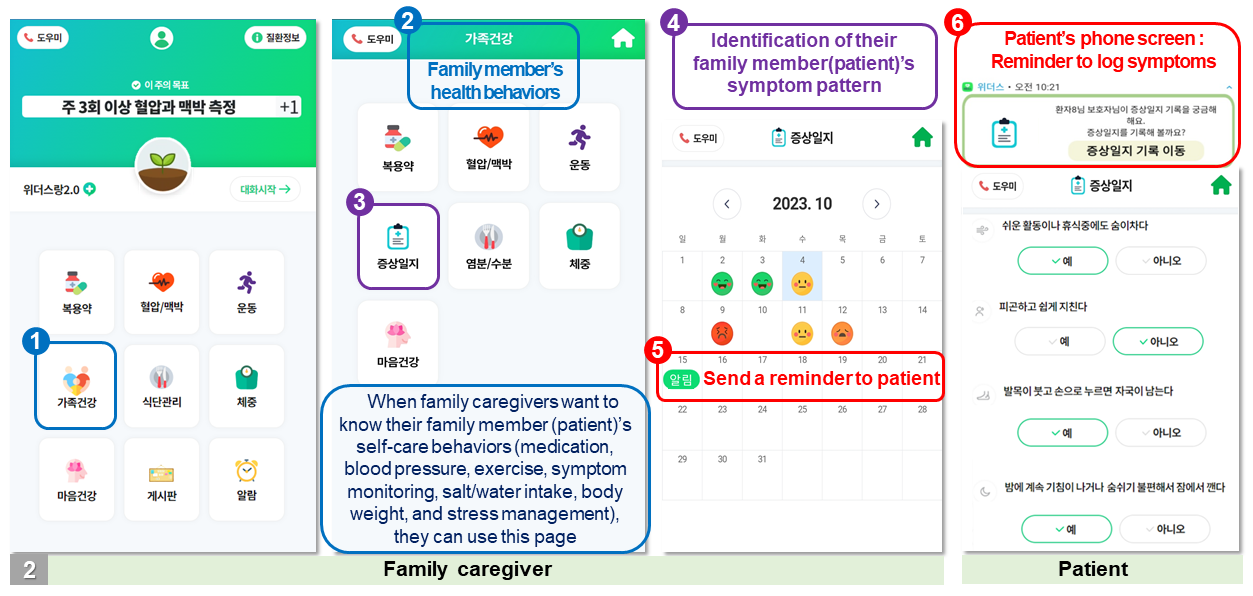


**Multimedia Appendix 1.** Patient and family caregiver interactions within dyadic WITHUS. [1-1. Patients’ main page, 1-2. Family caregivers’ main page]

WITHUS: Welcome to Interactive Text Messaging for Improving Heart Failure Self-Care Unified Supporters.
