# Supplementary figures and images for: Development and Evaluation of a Patient–Family Caregiver Dyad mHealth Intervention for Heart Failure Self-Care: Quasi-Experimental Study
Source: J Med Internet Res. 2025 Jun 16;27:e74922. doi: 10.2196/74922 (PMC12209723; doi:10.2196/74922)

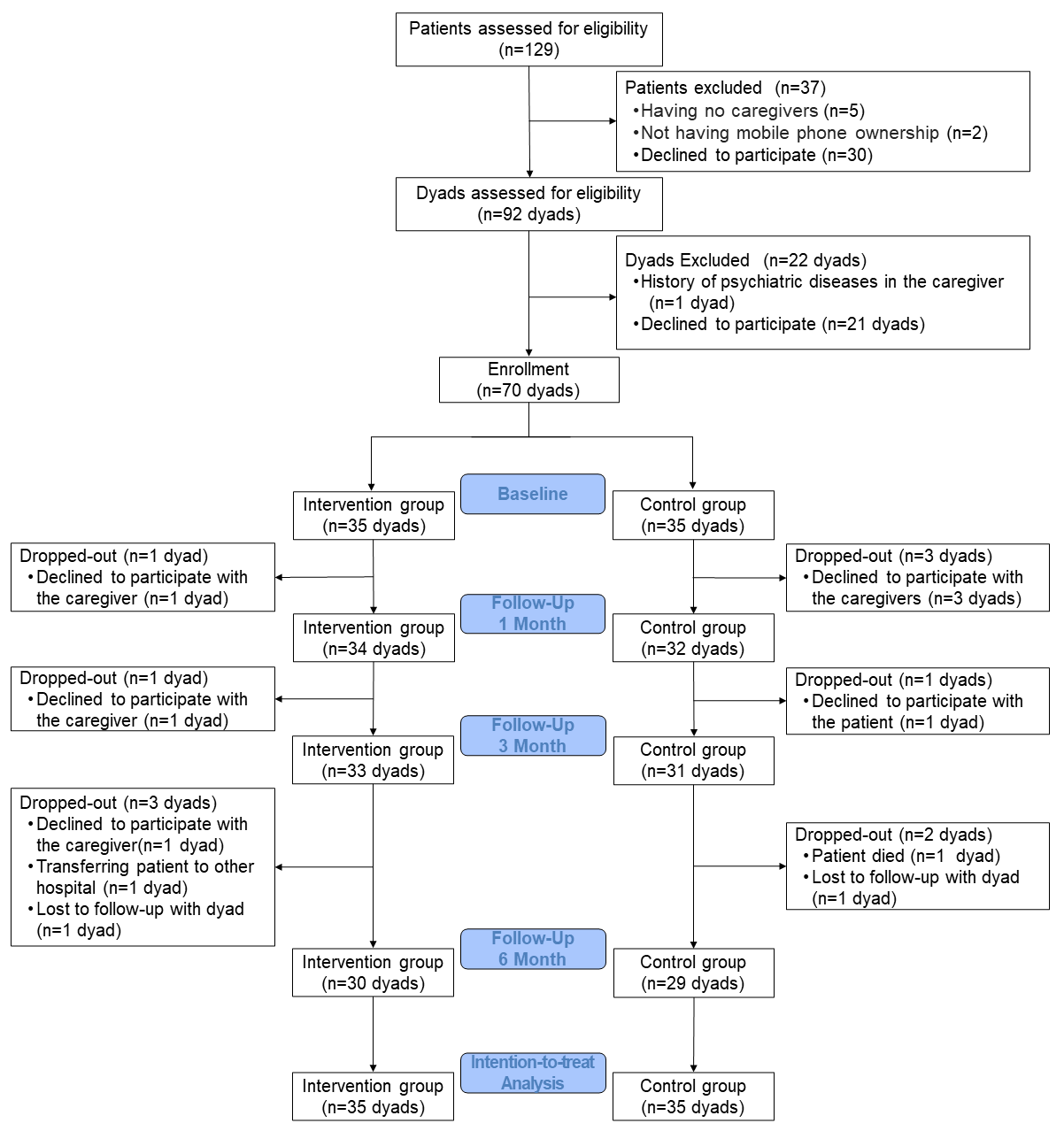


**Multimedia Appendix 2.** Flow chart of this study.

Supplement: Multimedia Appendix 2 [file jmir_v27i1e74922_app2.doc]
