# Supplementary material for: Development and Evaluation of a Patient–Family Caregiver Dyad mHealth Intervention for Heart Failure Self-Care: Quasi-Experimental Study
Source: J Med Internet Res. 2025 Jun 16;27:e74922. doi: 10.2196/74922 (PMC12209723; doi:10.2196/74922)
Supplement: Multimedia Appendix 3 [file jmir_v27i1e74922_app3.doc]

**Multimedia Appendix 3**. User version of the mobile application rating scale (uMARS) scores after a 24-week intervention (n=30).

| Item | | Patients | Family caregivers |
| --- | --- | --- | --- |
|  |  | Median (IQR) | Median (IQR) |
| *App quality (total)* | | 2.6 (2.5-2.7) | 3.9 (3.9-4.0) |
| Engagement (total) | | 3.2 (3.0-3.4) | 3.6 (3.6-3.9) |
|  | 1. Entertainment | 4.0 (3.0-4.0) | 3.0 (3.0-3.3) |
|  | 2. Interest | 4.0 (3.0-4.0) | 3.0 (3.0-3.3) |
|  | 3. Customization | 4.0 (3.3-4.0) | 4.0 (3.8-4.0) |
|  | 4. Interactivity | 4.0 (4.0-4.0) | 4.0 (4.0-4.0) |
|  | 5. Target group | 4.5 (4.0-5.0) | 4.0 (4.0-4.3) |
| Functionality (total) | | 4.4 (4.3-4.5) | 4.0 (4.0-4.3) |
|  | 6. Performance | 4.0 (4.0-5.0) | 4.0 (4.0-4.0) |
|  | 7. Ease of use | 5.0 (4.0-5.0) | 4.0 (4.0-4.0) |
|  | 8. Navigation | 4.0 (4.0-4.0) | 4.0 (4.0-4.0) |
|  | 9. Gestural design | 4.5 (4.0-5.0) | 4.0 (4.0-4.3) |
| Aesthetic (total) | | 4.0 (4.0-4.3) | 3.7 (3.7-4.0) |
|  | 10. Layout | 4.0 (4.0-4.8) | 4.0 (4.0-4.0) |
|  | 11. Graphics | 4.0 (4.0-4.0) | 4.0 (4.0-4.0) |
|  | 12. Visual appeal | 4.0 (4.0-4.0) | 3.0 (3.0-4.0) |
| Information (total) | | 3.3 (3.0-3.5) | 4.3 (4.3-4.3) |
|  | 13. Quality of information | 4.0 (4.0-4.0) | 4.0 (3.8-4.0) |
|  | 14. Quantity of information | 5.0 (5.0-5.0) | 5.0 (5.0-5.0) |
|  | 15. Visual information | 4.5 (4.0-5.0) | 4.0 (4.0-4.0) |
|  | 16. Credibility | 4.0 (4.0-5.0) | 4.0 (4.0-5.0) |
| *Subjective app quality* | |  | |
|  | 1. Recommend? | 3.0 (2.0-3.0) | 2.0 (2.0-3.0) |
|  | 2. Estimated frequency | 4.0 (3.0-4.0) | 3.0 (3.0-4.0) |
|  | 3. Would you pay? | 3.0 (2.0-3.0) | 2.0 (1.8-3.0) |
|  | 4. Overall rating | 4.0 (3.3-4.0) | 3.0 (3.0-4.0) |
| *Perceived impact* | |  | |
|  | 1. Awareness | 4.0 (4.0-5.0) | 4.0 (4.0-4.0) |
|  | 2. Knowledge | 4.0 (4.0-5.0) | 4.0 (4.0-4.0) |
|  | 3. Attitudes | 4.0 (4.0-5.0) | 4.0 (4.0-4.0) |
|  | 4. Intention to change | 4.0 (4.0-5.0) | 4.0 (4.0-4.0) |
|  | 5. Help seeking | 4.0 (4.0-5.0) | 4.0 (4.0-4.0) |
|  | 6. Behavior change | 4.5 (4.0-5.0) | 4.0 (4.0-4.3) |
