# Supplementary material for: Development and Evaluation of a Patient–Family Caregiver Dyad mHealth Intervention for Heart Failure Self-Care: Quasi-Experimental Study
Source: J Med Internet Res. 2025 Jun 16;27:e74922. doi: 10.2196/74922 (PMC12209723; doi:10.2196/74922)
Supplement: Multimedia Appendix 4 [file jmir_v27i1e74922_app4.doc]

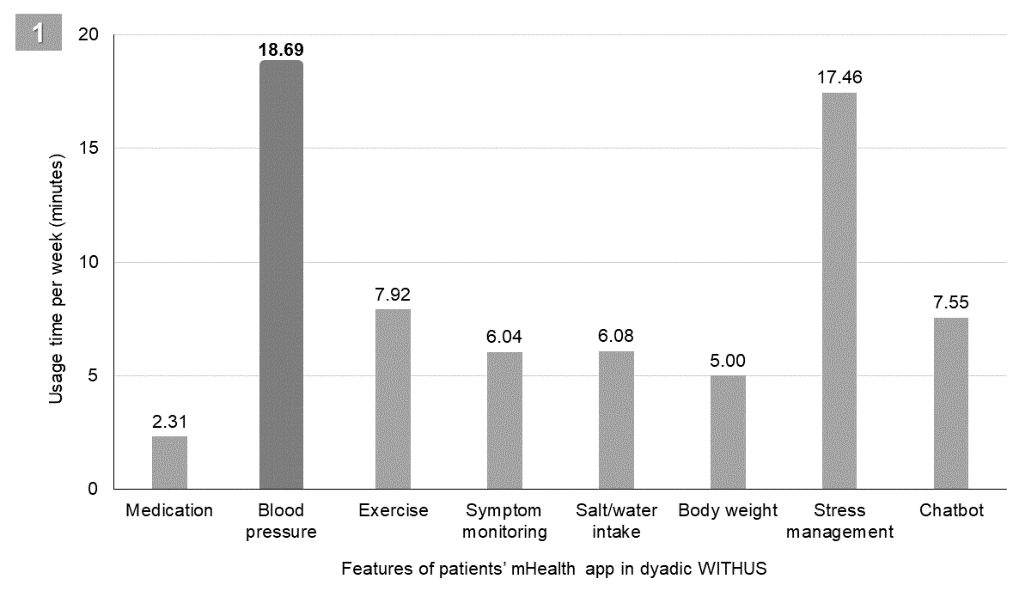

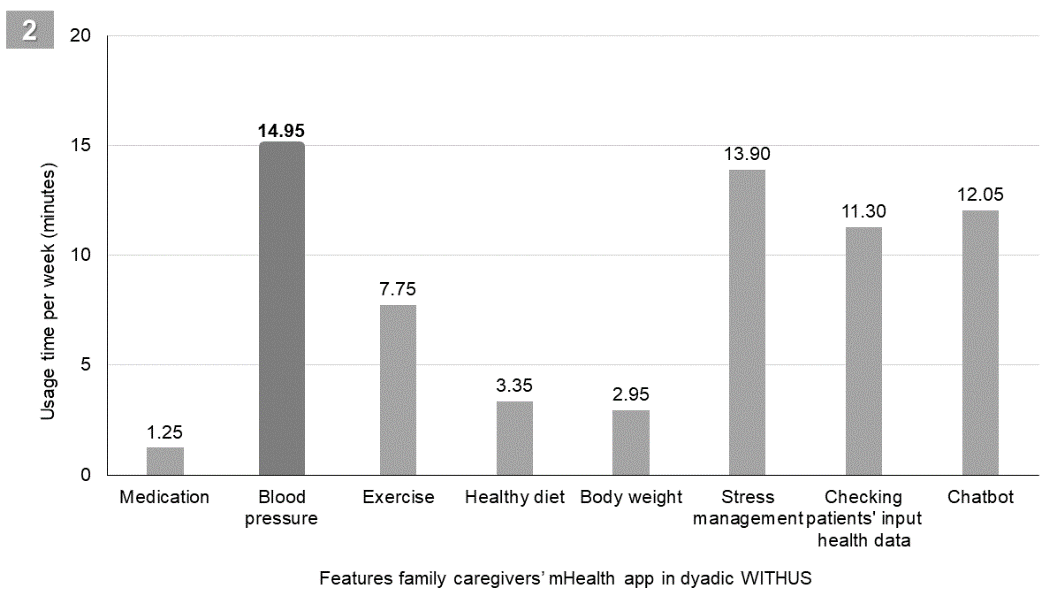


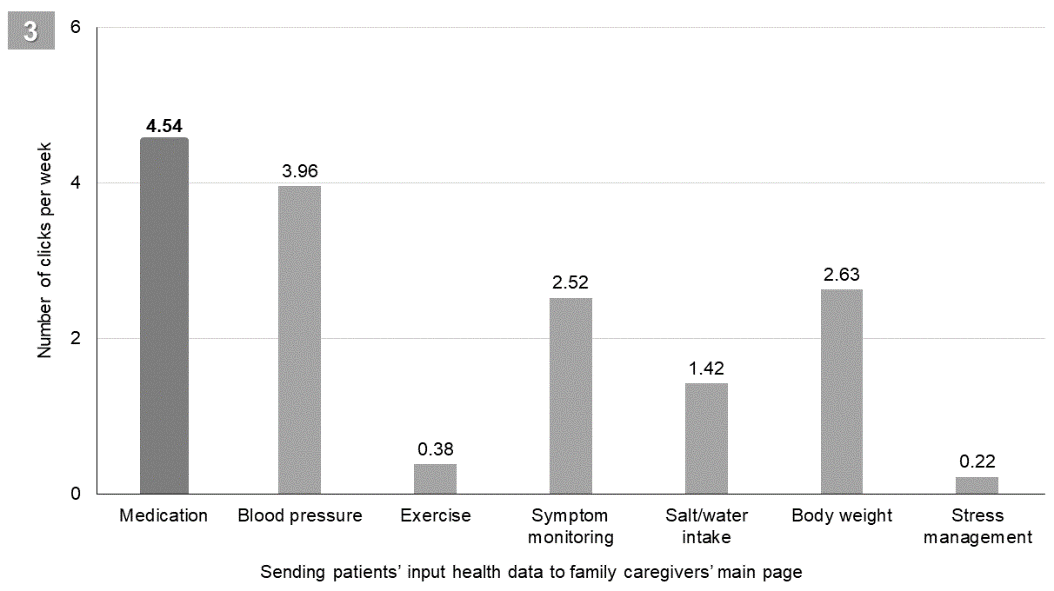

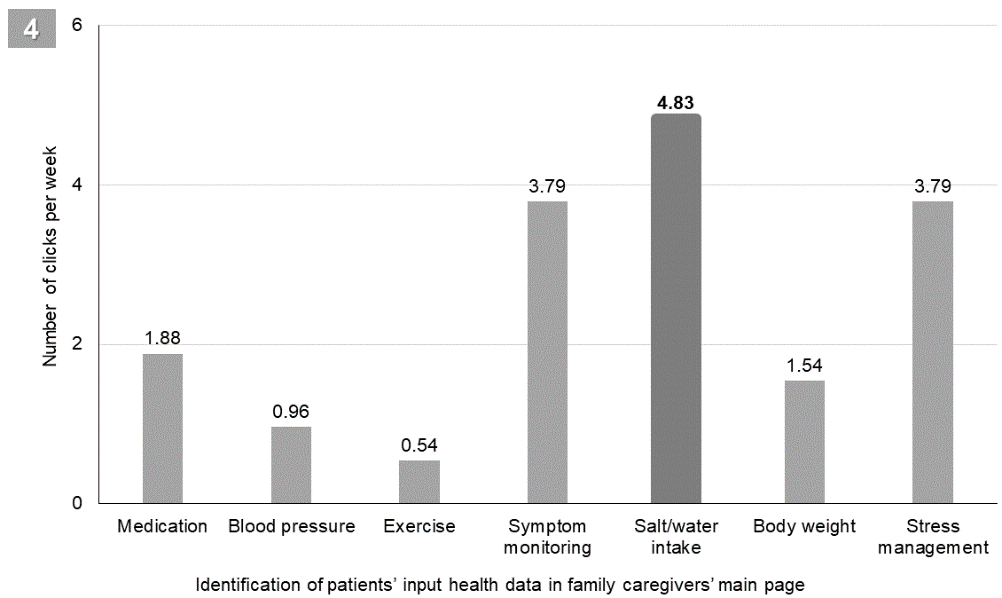


**Multimedia Appendix 4**. Usage time and number of clicks by features in dyadic WITHUS.

[4-1. Patients’ mHealth app, 4-2. Family caregivers’ mHealth app, 4-3. Sending patients’ input health data to family caregivers’ main page,
4-4. Identification of patients’ input health data in family caregivers’ main page]

WITHUS: Welcome to Interactive Text Messaging for Improving Heart Failure Self-Care Unified Supporters.
